# Keratinocyte Growth Factor Gene Delivery via Mesenchymal Stem Cells Protects against Lipopolysaccharide-Induced Acute Lung Injury in Mice

**DOI:** 10.1371/journal.pone.0083303

**Published:** 2013-12-18

**Authors:** Jie Chen, Chunsun Li, Xiaofang Gao, Chonghui Li, Zhixin Liang, Ling Yu, Yanqin Li, Xiaoyi Xiao, Liangan Chen

**Affiliations:** Department of Respiratory Medicine, Chinese People's Liberation Army General Hospital, Beijing, China; University of Illinois College of Medicine, United States of America

## Abstract

Acute lung injury (ALI) and acute respiratory distress syndrome (ARDS) are associated with high morbidity and mortality, and have no specific therapy. Keratinocyte growth factor (KGF) is a critical factor for pulmonary epithelial repair and acts via the stimulation of epithelial cell proliferation. Mesenchymal stem cells (MSCs) have been proved as good therapeutic vectors. Thus, we hypothesized that MSC-based KGF gene therapy would have beneficial effects on lipopolysaccharide(LPS)-induced lung injury. After two hours of intratracheal LPS administration to induce lung injury, mice received saline, MSCs alone, empty vector-engineered MSCs (MSCs-vec) or KGF-engineered MSCs (MSCs-kgf) via the tail vein. The MSCs-kgf could be detected in the recipient lungs and the level of KGF expression significantly increased in the MSCs-kgf mice. The MSC-mediated administration of KGF not only improved pulmonary microvascular permeability but also mediated a down-regulation of proinflammatory responses (reducing IL-1β and TNF-α) and an up-regulation of anti-inflammatory responses (increasing cytokine IL-10). Furthermore, the total severity scores of lung injury were significantly reduced in the MSCs-kgf group compared with the other three groups. The underlying mechanism of the protective effect of KGF on ALI may be attributed to the promotion of type II lung epithelial cell proliferation and the enhancement of surfactant synthesis. These findings suggest that MSCs-based KGF gene therapy may be a promising strategy for ALI treatment.

## Introduction

Acute lung injury (ALI) and acute respiratory distress syndrome (ARDS) are life-threatening conditions of acute respiratory failure, which are caused by direct or indirect injury to the lung [1]. Despite advances in therapeutic principles, ALI/ARDS remains a leading cause of morbidity and mortality (up to 30 to 40%) in critically ill patients [1], [2]. The disruption of alveolar epithelial integrity is a major contributor to increased permeability and alveolar flooding with protein-rich edema fluid, a hallmark of ALI/ARDS. However, pharmacological therapies that target the enhancement or restoration of lung epithelial cell functions have not yet been translated to effective clinical treatment options, and innovative therapies are urgently needed [3].

Keratinocyte growth factor (KGF), also known as fibroblast growth factor (FGF)-7, is a potent mitogenic factor for alveolar epithelial cells [4]. It has many characteristics that have been proved beneficial for ALI. For example, KGF can induce alveolar type II cell proliferation in vitro, stimulate surfactant synthesis, improve edema clearance and decrease apoptosis [5], [6]. However, the administration of exogenous rhKGF has so far demonstrated only limited effectiveness in a variety of animal models of lung injury induced by radiation [7], bleomycin [8] and acid aspiration [9]. Its clinical usefulness is still limited by its rapid degradation and short duration of action [10]. Thus, methods to ensure the sustained expression of KGF in the injured lung have important clinical applications.

Mesenchymal stem cells (MSCs) are adult stem cells that possess unique immunomodulatory and paracrine properties, which make them attractive for cell based therapy [11]. Previous studies have demonstrated that MSCs had beneficial effects on experimental models of ALI in both animals [12], [13] and human tissue [14]. Furthermore, MSCs have not only been used as therapeutic cells but also developed as the ideal cellular vehicles for gene delivery because they are self-renewable, easily expandable ex vivo and can engraft well [15], [16].

Cell-based gene therapy that uses a combination of cell and gene therapies has been proved successful in experimental models of pulmonary vascular disease, such as ALI/ARDS [17], [18] and pulmonary hypertension [19], [20]. This therapeutic strategy could improve the targeted expression of the transgene and overcome the shortcomings of a short protein half-life after injection. Therefore, this study aimed to investigate the ability of transplanted MSCs that stably overexpress the KGF gene to induce long-term epithelial-specific KGF production and improve the prognosis of ALI in an LPS-induced lung injury mouse model.

We report that MSC-based KGF gene therapy can deliver KGF to the injured lung tissues and attenuates LPS-induced ALI mice. Moreover, we also demonstrate that bone MSCs expressing KGF in the lung can induce proliferation of lung epithelial cells and promote the secretion of surface proteins.

## Materials and Methods

### Isolation, culture and characterization of mesenchymal stem cells

C57BL/6 male mice were obtained from the Laboratory Animal Center of the Chinese People's liberation army (PLA) general hospital (Beijing, China). All animals were raised and used in accordance with the National Institutes of Health Guidelines on the Use of Laboratory Animals. All experiments and procedures were approved by the Committee on the Ethics of Animal Care and Use of Chinese PLA general hospital (Permit Number: 2011-X-25). All surgeries were performed under sodium pentobarbital anesthesia, and all efforts were made to minimize suffering.

MSCs were obtained from 2 week-old male C57BL/6 mice. The bone marrow was collected by flushing the tibiae and femurs using Iscove's modified Dulbecco's medium (IMDM, Hyclone). The cells were washed, re-suspended and then placed in IMDM containing 10% fetal bovine serum, 1% L-glutamine and 1% penicillin-streptomycin (Gibco, MD, USA). After 4 days of cultivation, the non-adherent cells were removed from the culture by changing the medium, while adherent cells were sub-cultured then subsequently propagated. MSCs in passages 4–6 (p4–p6) were used for the experiments. Adipogenic and osteogenic differentiation assays were performed in six-well plates according to previously described methods [12]. We also confirmed the presence of MSCs based on previously accepted markers [21], [22], i.e., the presence of stromal cell markers, such as CD29 and CD90, and the absence of hematopoietic markers, such as CD31, CD34, and CD45. The antibodies were obtained from the following sources: anti mouse CD31, CD34, CD90 and rat IgG2a kappa isotype control antibody; anti mouse CD45 and rat Ig2b kappa isotype control antibody; anti mouse CD29 and American Hamster IgG isotype control antibody (ebioscience, San Diego, CA). A flow cytometry analysis was performed according to standard procedures.

### Generation of lentiviral vectors and transduction into MSCs

The KGF gene was acquired by PCR from a plasmid, a kind gift from Dr. JA Whitsett (Cincinnati Children's Hospital Medical Center, Cincinnati, USA). The KGF gene was then cloned into the multi-cloning site of a retroviral vector plasmid pLV-UbC-IRES2-EGFP (sbo-bio). Recombinant lentiviruses were produced in 293T cells following the co-transfection of pLV-UbC-IRES2-EGFP or pLV-UbC-KGF-IRES2-EGFP and packaging plasmids pCD/NL-BH and pLTR-G. High titer recombinant lentiviral vectors with KGF or eGFP were also generated. MSCs (1×10^5^) from p4 were transduced with lentiviral vectors at a multiplicity of infection (MOI) of 20. After one week, MSCs carrying eGFP (MSCs-vec) or MSCs carrying both eGFP and KGF genes (MSCs–KGF) were examined using a fluorescent microscope and then harvested. The overexpression of the KGF gene was detected by both real time PCR and western blotting. The non-transduced and transduced MSCs were placed in serum-free culture medium. At the beginning and after day 3 and day 7, the levels of KGF protein in the conditioned medium were tested by ELISA (R&D systems). Adipogenic and osteogenic differentiation assays were also performed on the transduced MSCs.

### Murine model of LPS-induced ALI

Eight week-old inbred male C57BL/6 mice were anaesthetized with pentobarbital (90 mg/kg). ALI was then induced via the intratracheal administration of LPS from Escherichia coli O55:B5 (E. coli 055:B5; Sigma) at a dosage of 10 mg/kg as described previously [12]. Two hours after LPS administration, normal saline (NS group), MSCs alone (MSCs group), empty vector-engineered MSCs (MSCs-vec) or KGF-engineered MSCs (MSCs-kgf) (5×10^5^ cells, 200 µL total volume) were slowly infused via the tail vein. A fluorescent cell tracker, CM-Dil (Molecular Probes, Carlsbad, CA), was used to label MSCs immediately before transplantation, and the eGFP gene was used to trace the engineered MSCs. The mice were humanely killed at 0 hours (before LPS administration), 6 hours, 24 hours, 72 hours or 168 hours after MSC infusion to collect tissues for analysis. We used two measurement sets of animals to accurately measure the wet/dry weight ratio. In one set of animals (n = 5 per group), the lungs were lavaged back and forth three times with 1 mL of cold saline. The bronchoalveolar lavage fluid (BALF) was collected and then centrifuged at 500 g for 10 min at 4°C. The supernatants were collected and stored at −20°C until testing. The cell pellet was resuspended in 1 mL balanced salt solution. A cell smear was generated, and the cells were visualized using Wright–Giemsa staining. A differential of the white blood cells was then obtained by counting 100 cells from a representative portion of the slide [23]. The pulmonary circulation was flushed with 1 mL cold (4°C) phosphate-buffered saline (PBS) via the right ventricle injection to remove blood-borne elements and plasma. The lungs were snap-frozen and later processed to obtain lung homogenates according to a previously published protocol [17]. In another set of animals (n = 5 per group), the blood was collected with a heparinized needle via cardiac puncture and later centrifuged at 2,000 g to obtain a plasma sample. The right upper lobe was excised and weighed immediately, and the lung tissue was then placed in a drying oven at 55°C for 24 h to subsequently determine the dry weights. The final lung wet-to-dry ratio was calculated as described in previous publications [24]. The left lower lobe was collected to prepare frozen sections, and the rest was fixed by formalin and then prepared for paraffin-embedded sections. In addition, 40 mice were randomly divided into the above 4 groups (n = 10 in each group). Survival was then assessed for up to 168 hours. Throughout this time period, the mice had free access to water and food, and their rectal temperature was carefully monitored. Mice with a low body temperature (<30°C) were considered to be close to death and were killed by anesthetic overdose [5].

### Assessment of BAL protein, myeloperoxidase (MPO) activity and cytokines

The total protein in BALF was quantified using the bicinchoninic acid (BCA) method as provided by the manufacturer (Pierce, Rockford, IL, USA). The MPO activity in the lung tissue was assayed as previously described standard methods [25]. The TNF-α, IL-1β and IL-10 levels in BALF and plasma samples were measured by ELISA (R&D Systems) according to the manufacturer's protocol.

### Real-time PCR of KGF, SPA, SPB, SPC, SPD

Total RNA was prepared with TRIzol Reagent following manufacturer's instructions (GibcoBRL, Life Technologies). RNA was transcribed using Superscript III reverse transcriptase (Invitrogen) and oligo-(dT). Real time RT-PCR was conducted using the Platinum SYBR Green qPCR SuperMix UDG (Takara Bio) on an iCycler iQ real-time RT–PCR system (Bio-Rad, USA). All primers were murine in origine and defined as follows: β-actin (Forward 5′-CATCCGTAAAGACCTCTATGCCAAC-3′; Reverse 5′-ATGGAGCCACCGATCCACA-3′), KGF (Forward 5′- TGGTACCTGAGGATTGACAAACGA-3′; Reverse 5′- CCTTTGATTGCCACAATTCCAAC-3′), SPA (Forward 5′- CTCGGAGGCAGACATCCACA-3′; Reverse 5′-TGATGCCAGCAACAACAGTCAA-3′), SPB (Forward 5′- GAGTGTGCACAAGGCCCTCA-3′; Reverse 5′- CCTCACACTCTTGGCACAGGTC-3′), SPC (Forward 5′-CATCATGAAGATGGCTCCAGAGA-3′; Reverse 5′- ACACAGGGTGCTCACAGCAAG-3′), SPD (Forward 5′-CCTCAAGGCAAACCAGGTCCTA-3′; Reverse 5′-TGCATGCCAGGAGCACCTAC-3′). The cycling conditions were as follows: 95°C for 10 min, 40 cycles of 94°C for 15 s, 54°C for 30 s, and 72°C for 30 s, followed by 5 min extension at 72°C and holding at 4°C. The fold changes were calculated using the delta-delta Ct method according to a previous report [26]. All reactions were run in triplicate.

### KGF Quantification by ELISA and Western blotting

The KGF protein in the lung homogenate or plasma was quantified by ELISA according to the manufacturer's instructions (R&D systems). A total of 40 µg of reduced, denatured MSCs proteins was separated on a 10% denaturing SDS polyacrylamide gel and transferred to a polyvinylidene difluoride membrane (Millipore). The membrane was saturated for 1 h at room temperature in PBS/0.05% Tween 20 supplemented with different concentrations of nonfat dried milk (Sigma-Aldrich). The membrane was then exposed to rabbit polyclonal primary Abs (Santa Cruz) for mouse KGF at a 1/200 dilution overnight at 4°C. The membrane was washed and then incubated with a goat anti-rabbit HRP-labeled Ab (GE Healthcare) at a 1/4000 dilution for 30 min. The protein bands were subsequently visualized with an ECL kit (Amersham Biosciences).

### Histopathological examination and immunohistochemistry

Frozen lung tissues were sectioned at a thickness of 20 µm by cryostat (Leica, Wetzlar, Germany) and then placed onto uncoated glass slides. The total cell nuclei of the tissues were stained with 4′,6-diamidino-2-phenylindole (DAPI) (Sigma-Aldrich). Finally, fluorescence images were acquired using a confocal laser scanning microscope (TCS SL; Leica, Heidelberg, Germany). The paraffin-embedded lungs were sectioned at a thickness of 5 µm and stained with hematoxylin and eosin (H&E). The images were evaluated by an investigator who was blinded to the identity of the slides to determine the lung injury score following a previously published scoring system [27]. After de-paraffinization, the paraffin-embedded lung sections were incubated overnight with anti-GFP (Bioss 1∶300), anti-SPC (Santa cruz 1∶200) and anti-PCNA (Santa cruz 1∶25), followed by an incubation with goat anti-rabbit (GFP and SPC) or anti-mouse (PCNA) IgG conjugated to horseradish peroxidase (Santa cruz). 3,3-diaminobenzidine (DAB; Pierce) was used as the chromogenic substrate. To quantify the persistence of MSCs *in vivo*, the number of cells that were positive for GFP staining was determined from 20 randomly selected areas per high-power field (magnification, ×400) with the Scion Image software.

### Statistical analysis

The results are presented as the mean±SD. Multiple groups were compared with a Tukey-Kramer *post hoc* test after an analysis of variance (ANOVA). Two continuous variables were compared using Student's t-test. The survival curves were derived with the Kaplan–Meier method and compared by log-rank tests. A *P* value <0.05 was considered statistically significant.

## Results

### Characterization of MSCs and transduction efficiency with lentivirus vector

**Figure 1:**
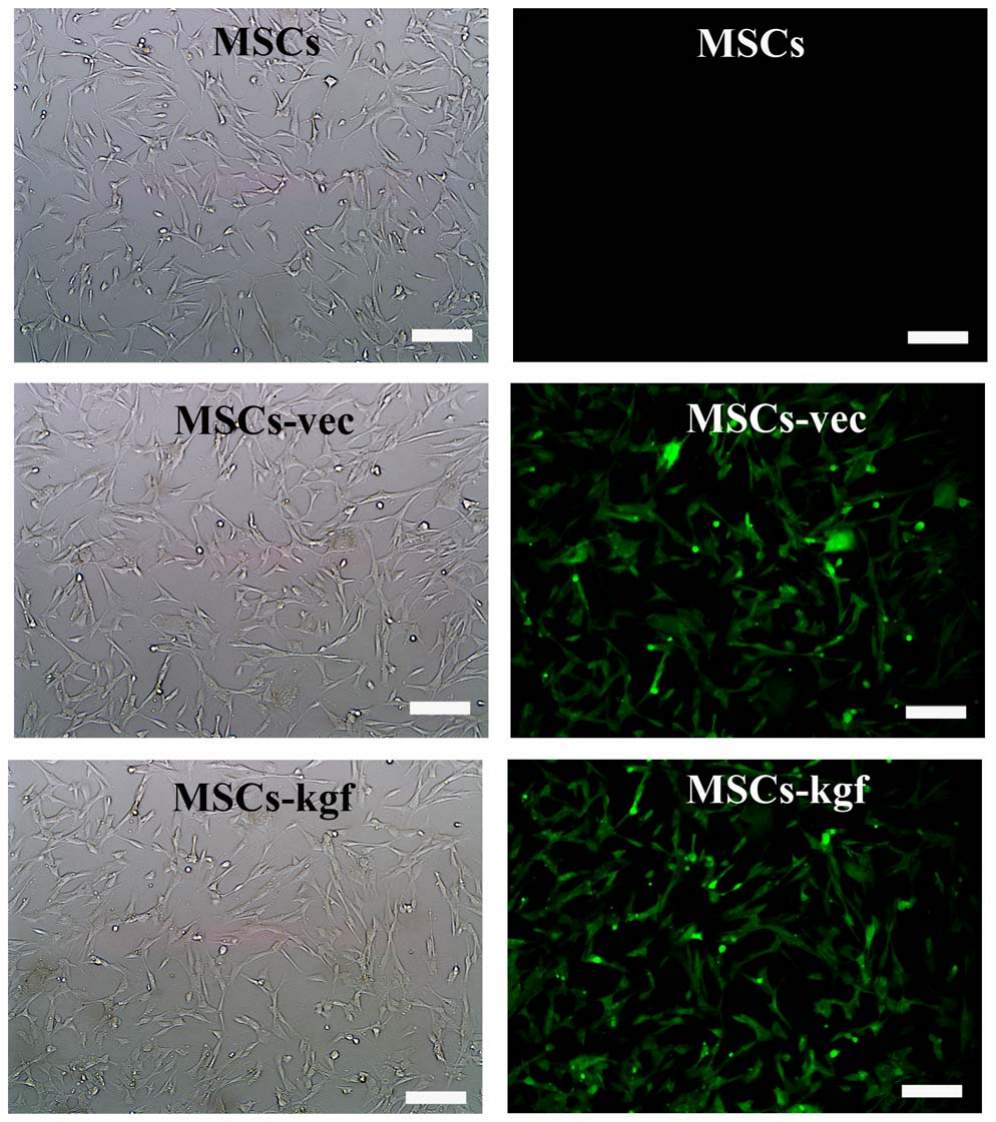
Transduction of KGF into MSCs using a lentiviral vector. The expression of GFP in MSCs was detected at day 7 after vec–eGFP or KGF–eGFP transduction under both white light microscopy (left) and fluorescence microscopy (right); (×200, Scale bar =  50 µm).

**Figure 2:**
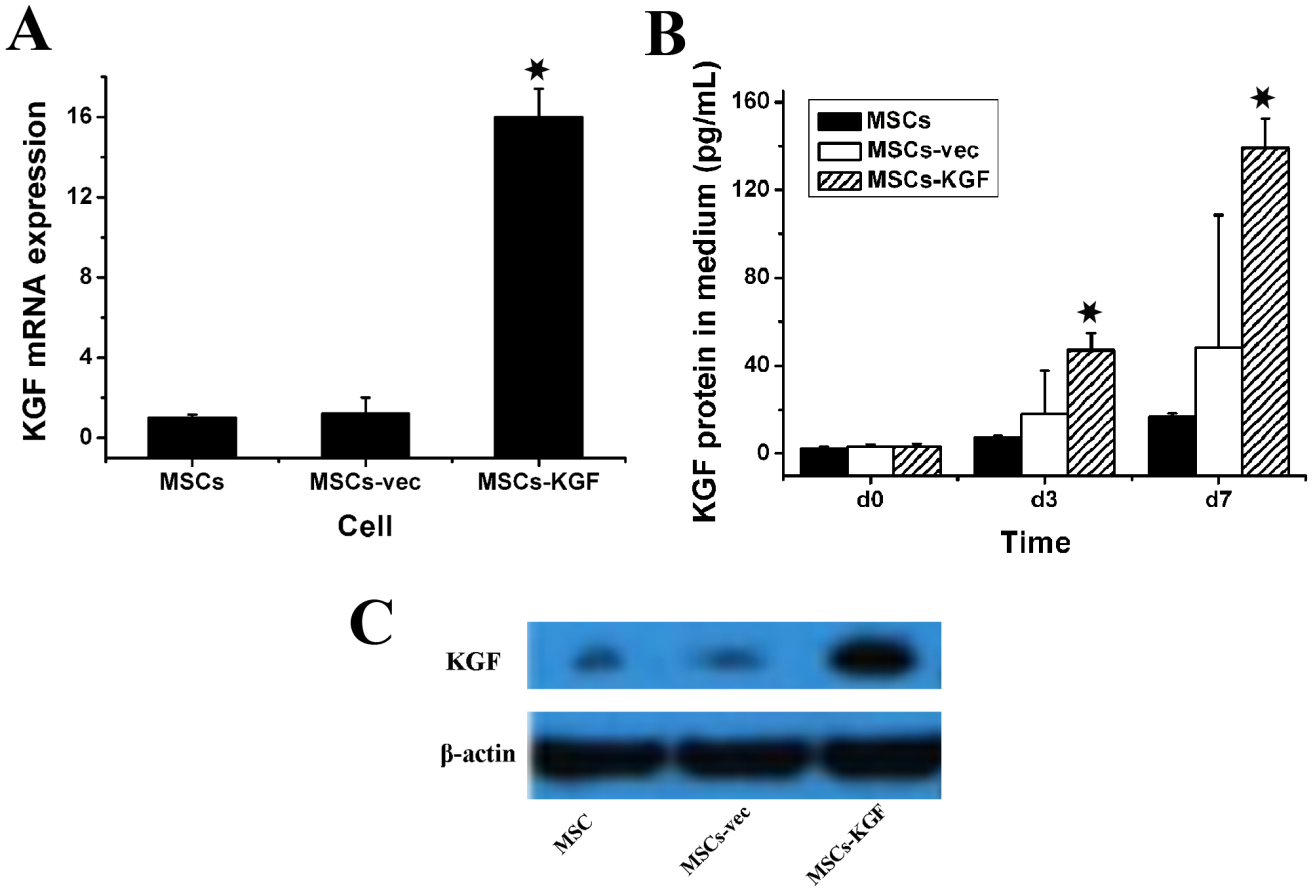
The KGF expression in *vivo*. (A) At a MOI = 20, KGF mRNA expression at day 7 after lentiviral vector transduction in the MSCs-KGF was approximately 16 times that of the MSCs-vec and MSCs alone as detected by real-time PCR. The data are expressed as the mean±SD, *p<0.01; (B) At a MOI = 20, KGF protein expression was detected by western blot at day 7 after lentiviral vector transduction (lane 1 MSCs, lane 2 MSCs-vec, lane 3 MSCs-KGF); (C) At a MOI = 20, the levels of KGF protein in medium in MSCs-KGF group were significantly higher than that in the MSCs-vec group and MSCs group at day 3 and day 7. Data were expressed as the mean±SD, *p<0.01.

Most of the MSCs were spindle-like in shape and adhered to the plastic tissue culture medium (Figure S1.A). The multipotent differentiation capacity of the MSCs was confirmed by their differentiation into adipocytes and osteoblasts. In adipogenic cultures, the intracellular accumulation of lipid droplets was stained purple with Oil Red O, which indicated adipogenesis (Figure S1.B). In osteogenic cultures, mineralized nodule-like structures were stained with alizarin red, which showed calcium deposition (Figure S1.C). A flow cytometric analysis (Figure S1.D) indicated that MSCs were positive for the mesenchymal stem cell markers CD29 and CD90 and negative for the hematopoietic lineage markers CD45 and CD34. These results also revealed that cultured MSCs were homogeneous and did not contain endothelial progenitor cells lineages (negative for CD31), which is consistent with previous reports [21]. After expansion over five passages, the MSCs were stably transduced with our lentivirus that expressed GFP or both GFP and KGF at different multiplicities of infection (MOI). An MOI of 20 was used for further experiments. The efficiency of transduction was as high as 90% at day seven (Figure 1). In addition, the phenotype of MSCs was not changed by the transduction processes. The MSCs-KGF was examined for KGF expression using real-time PCR and Western blots. Real-time PCR demonstrated that the level of KGF mRNA in the MSCs-KGF was increased approximately 15-fold (Figure 2.A, *p*<0.01 compared to MSCs-vec or MSCs alone). The KGF protein levels in the medium of the MSCs-KGF group were also higher than those of the control groups after MSC transduction 3 days and 7 days (Figure 2.B, *p*<0.01 compared to MSCs-vec or MSCs alone). Similarly, the Western blots showed that KGF protein expression in the MSCs-KGF was higher than that in the MSCs-vec or MSCs alone (Figure 2.C).

### Detection of transplanted MSCs in injured lung

**Figure 3:**
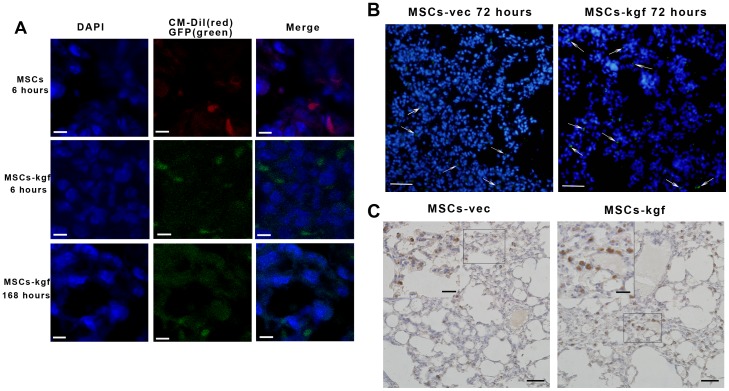
Detection of transplanted MSCs in injured lungs. (A) Non-transduced MSCs (labeled with CM-Dil, red) and KGF and GFP genes transduced MSCs (green) were observed in frozen lung sections from the MSCs group and MSCs-KGF group mice respectively. All images were obtained using a confocal laser scanning microscope with a 60× objective (Scale bar =  5 µm). (B) GFP gene transduced MSCs and KGF and GFP genes transduced MSCs were observed in frozen lung sections from the MSCs-vec group and MSCs-KGF group mice sacrificed at 72 hours respectively (green, white arrow). (×200, Scale bar =  50 µm). (C) Immunohistochemistry of GFP in lungs showed that GFP+ cells were more frequent in the MSCs-KGF group than in the MSCs-vec group (Scale bar =  50 µm; Inserts, scale bar =  20 µm).

The eGFP-positive cells were observed in both frozen and paraffin-embedded lung sections of the mice treated with MSCs–KGF or MSCs (Figure 3). To better observe the MSCs retention in mice, the total cell nuclei of the tissues were stained with DAPI, which resulted in blue fluorescence (Figure 3.A,B). CM-DiL-labeled cells and eGFP-positive cells were observed in the mice treated with MSCs or MSCs-kgf at 6 hours (Figure 3.A). On average, 41.2% of injected MSCs were found in the MSCs-kgf group compared to 38.5% in the MSCs-vec group at 6 hours, although the difference was not statistically significant (Figure S2). Irrespective of lung injury, most of the MSCs were lost from the lung after 7 days (less than 3%, Figure 3.A and Figure S2). Figure 3.B shows the overall extent of MSCs retention in the lung tissues at 72 hours after administration. Figure 3.C shows that GFP^+^ cells were clearly distributed in the lung alveolar epithelium. In addition, the number of GFP+ cells was much higher in the severely injured areas compared to mildly injured areas.

### Analysis of KGF expression in *vivo* after MSCs-kgf treatment

**Figure 4:**
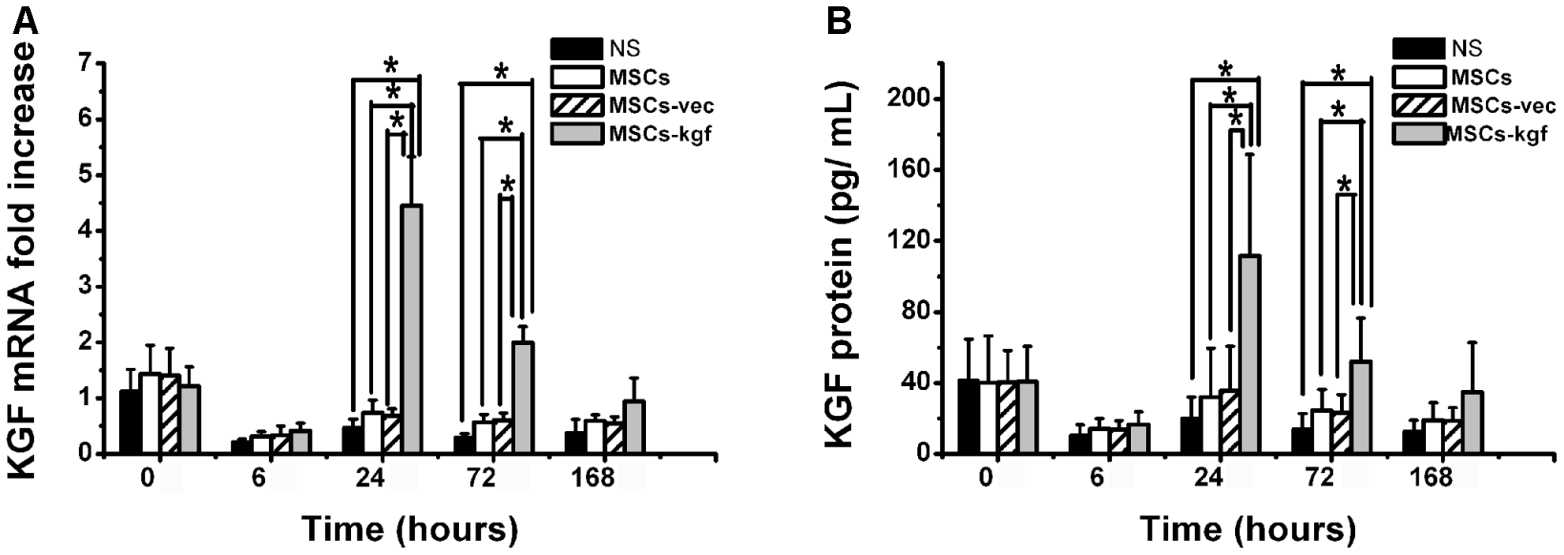
Analysis of KGF expressions in vivo after MSCs-KGF treatment. (A) The change in the total KGF mRNA levels was analyzed using real-time PCR, and the values were normalized to that of the NS group at 0 hours before LPS administration. Group comparisons were analyzed by a one-way ANOVA with a Tukey-Kramer *post hoc* test. n = 5 per group. *p<0.05. (B) The total KGF protein in the lung tissues was analyzed using ELISA. Group comparisons were analyzed by a one-way ANOVA with a Tukey-Kramer *post hoc* test. The data are expressed as the mean±SD. n = 5 per group. *p<0.05

The change in the total KGF mRNA levels was analyzed using real-time PCR, and the values were normalized to that of the NS group before LPS administration. The total KGF mRNA levels of the MSCs-kgf group were significantly elevated compared with the other three control groups at both 24 hours and 72 hours (Figure 4.A, *p*<0.05). Moreover, the total protein level of KGF in the lung tissues of the MSCs-kgf group displayed a similar tendency (Figure 4.B).

### Effect on BALF protein and lung wet/dry ratio

**Figure 5:**
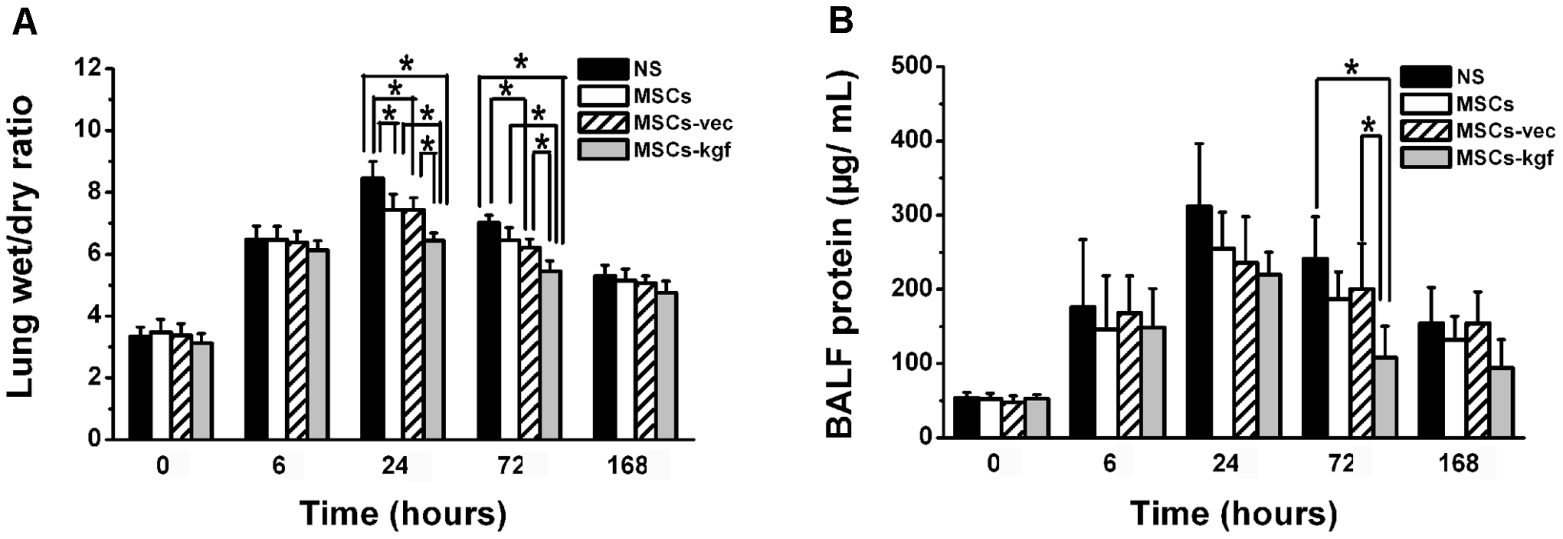
Effect of MSCs-KGF on LPS-Induced ALI Permeability. Therapeutic efficacy on LPS-induced ALI permeability was assessed by measuring the BALF protein and lung wet/dry ratio. (A) Lung wet/dry ratio group comparisons were analyzed by a one-way ANOVA with a Tukey-Kramer post hoc test. (B) Total protein concentration in the BALF was measured using the bicinchoninic acid (BCA) method. Comparisons were analyzed by a one-way ANOVA with Tukey-Kramer post hoc test. The data are expressed as the mean±SD. n = 5 per group. *p<0.05.

Pulmonary edema is a hallmark of ALI. The treatment of animals with MSCs or MSCs-vec significantly attenuated the increase in the lung wet/dry ratio at 24 and 72 hours after LPS administration (*p*<0.05 compared to NS group). Treatment with MSCs-kgf further attenuated the increase in the lung wet/dry ratio (*p*<0.01 compared to NS group Figure 5.A). BALF protein was a marker to evaluate the integrity of the alveolar–capillary membrane barrier and assess pulmonary vascular leakage in ALI. The total protein of in the BALF in the MSCs-kgf group was much lower than those in the control groups at 72 hours after LPS instillation (*p*<0.01 compared to NS group, *p*<0.05 compared to MSCs-vec group, Figure 5.B).

### Assessment of lung inflammation after MSCs-kgf administration

**Figure 6:**
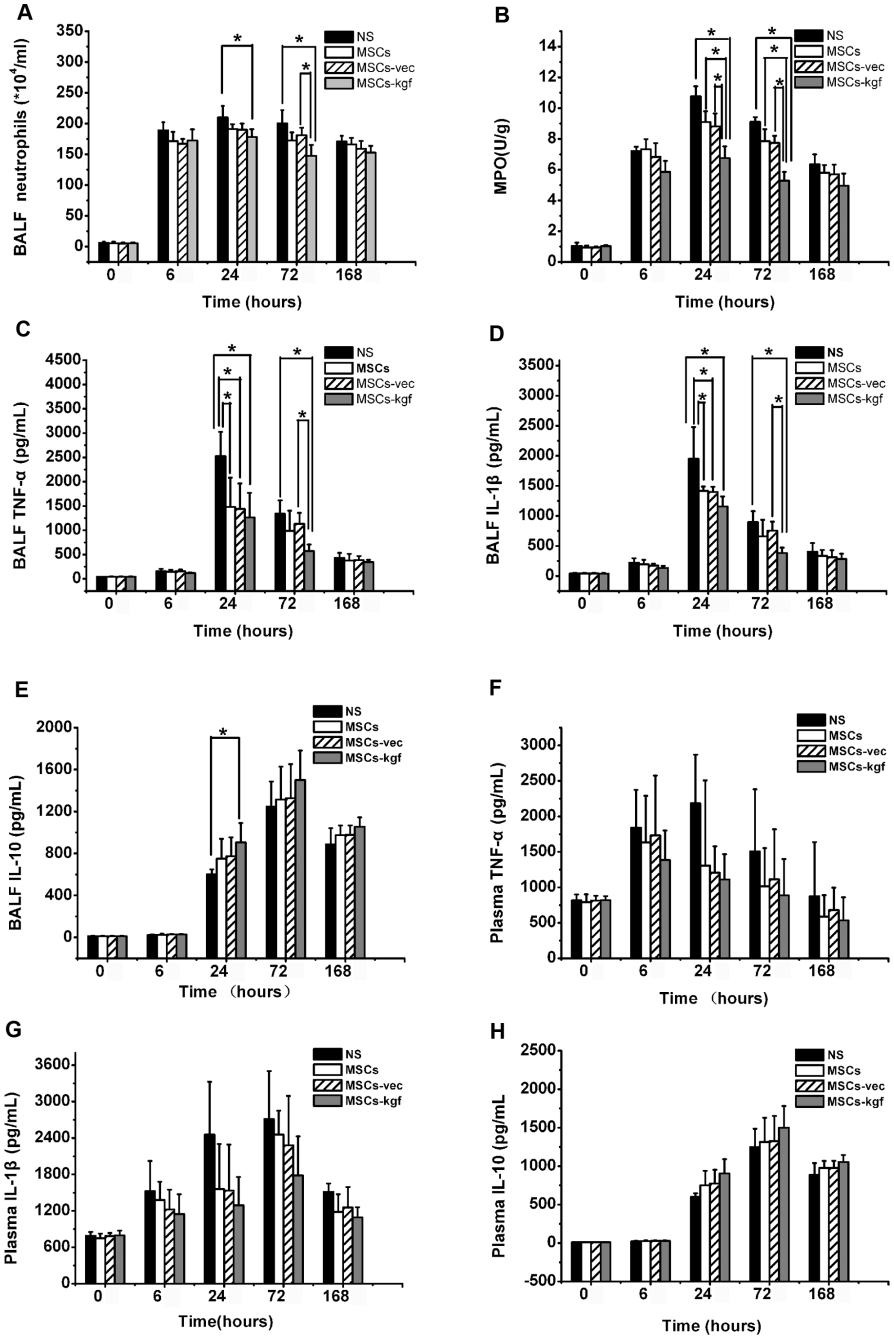
Assessment of lung inflammation after MSCs-KGF administration. (A) BALF neutrophils count; (B) MPO activity; (C) Levels of TNFα in BALF; (D) Levels of IL-1β in BALF; (E) Levels of IL-10 in BALF; (F) Levels of TNFα in plasma; (G) Levels of IL-1β in plasma; (H) Levels of IL-10 in plasma from 20 randomly selected areas per high-power field (magnification, ×400) with Scion Image software. *p<0.05.

LPS-challenged mice showed a significant reduction in the BALF neutrophil count in the MSCs-kgf group at 24 hours (Figure 6.A, *p*<0.05 compared to NS group) and at 72 hours (Figure 6.A, *p*<0.01 compared to NS group, *p*<0.05 compared to MSCs-vec group). Similarly, the MPO activity was reduced in the MSCs-kgf treatment group at 24 hours and 72 hours (Figure 6.B, *p*<0.01 compared to NS group, *p*<0.05 compared to MSCs group or MSCs-vec group). The levels of pro-inflammatory cytokines (TNF-α and IL-1β) were elevated in BALF in response to LPS. They were significantly reduced by treatment with MSCs or MSCs-kgf at 24 hours (Figure 6.C and Figure 6.D, *p*<0.05 compared to NS group). Moreover, treatment with MSCs-kgf further reduced the BALF pro-inflammatory cytokines compared to treatment with MSCs-vec at 72 hours (Figure 6.C and Figure 6.D, *p*<0.05). LPS administration also increased the level of the anti-inflammatory cytokine IL-10 in BALF. In addition, the increase in the LPS-induced anti-inflammatory cytokine (IL-10) was significantly different between the MSCs–kgf and the NS groups at 24 hours (Figure 6.E, *p*<0.05). Similar trends were observed in the plasma, although the differences were not statistically significant between the four groups (Figure 6.F, G, H).

### MSCs-kgf administration improves lung histopathology and survival analysis

**Figure 7:**
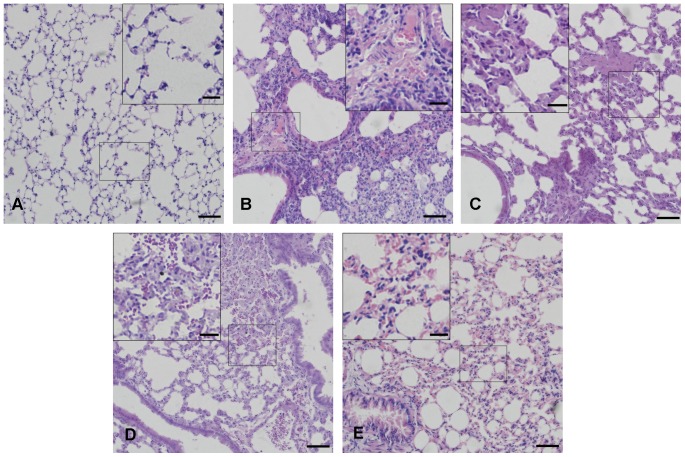
MSCs-KGF administration improve lung histopathology. Representative images of hematoxylin and eosin-stained lung sections from four experimental groups. (A) HE staining of lung sections before administration of LPS showed no obvious lesion in the lung tissues. (B) The lung interstitium of the NS group showed reduced cavities of pulmonary alveolus, interstitial edema and hemorrhage. (C) Lung injury of the MSCs group was improved but still apparent compared with that of the NS group. (D) Lung injury of the MSCs-vec group was similar to that of the MSCs group. (E) Lung injury of the MSCs-KGF group was remarkably improved compared with that of the NS group. (Scale bar =  100 µm; Inserts, scale bar =  20 µm).

**Figure 8:**
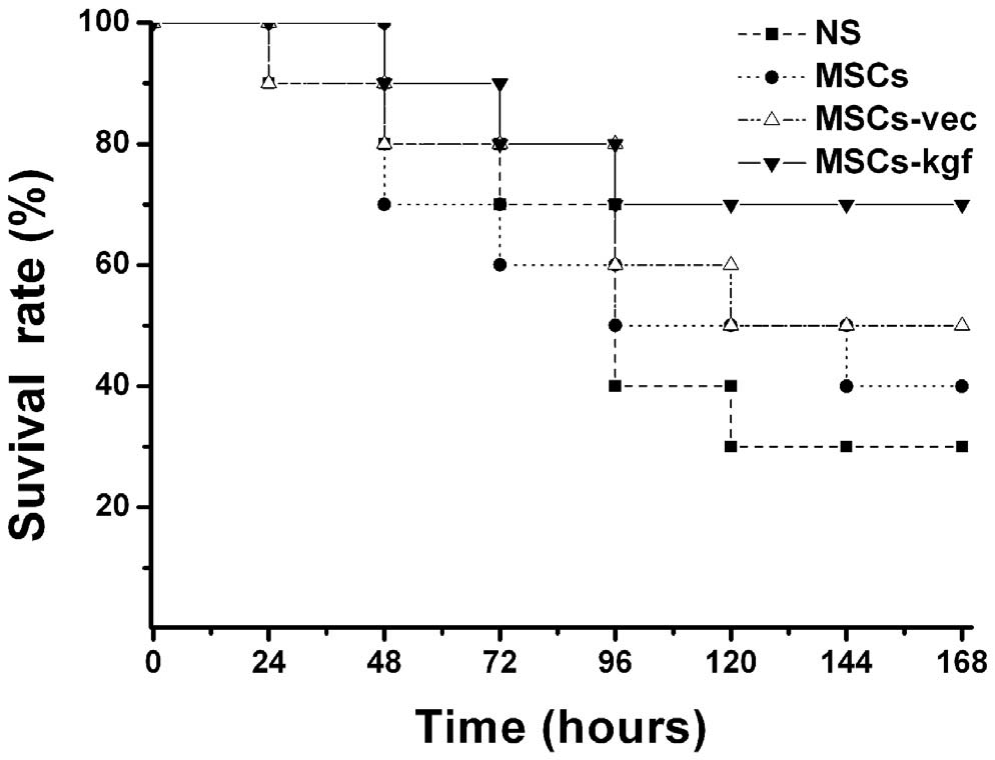
Kaplan-Meier survival curves. After 2×10^5^ cells of MSCs or MSCs-vec or MSCs-kgf were transplanted in ALI mice. NS group received NS instead of cell transplantation. All the mice were observed for 168 hours after cell transplantation. (n = 10/group).

The HE staining of lung sections before the administration of LPS showed no obvious lesions in the lung tissues (Figure 7.A). In the NS group, the lung sections displayed extensive morphological lung damage, manifested by the infiltration of numerous polymorphonuclear leukocytes and macrophages in the interstitial spaces, intra-alveolar and interstitial patchy congestion and hemorrhage, interalveolar septal thickening and interstitial edema (Figure 7.B). The administration of MSCs improved the lung histopathology, which was more apparent in mice treated by MSCs-kgf (Figure 7.C, D, E). The severity of lung injury was also scored by the histopathology score system. Compared with the NS group, the lung injury scores were significantly reduced in the MSCs–kgf group at 72 hours after LPS administration (Table 1). The Kaplan-Meier survival curves showed that mice in the NS group, MSCs group and MSCs-vec group began to die after 24 hours, and the survival rate progressively decreased (Figure 8). The survival rate at 168 hours in the MSCs-kgf treatment group was higher than that of the control groups, although the difference was not statistically significant (*p*>0.05).

**Table 1:** Severity scorings of lung injury of the four groups of mice.

Time(hours)	NS	MSCs	MSCs-vec	MSCs-KGF
6	5.86±1.06	5.60±1.19	4.67±1.47	5.70±1.25
24	8.16±1.82	7.36±1.52	6.77±1.75	6.67±1.94
72	12.73±2.42	10.68±2.53	10.87±3.06	7.9±2.14*
168	10.37±1.98	8.49±1.62	8.81±1.94	6.61±1.12

Data are shown as the mean±SD, n = 5 for each point. *p<0.05 compared with the NS group.

### Analysis of SP mRNA expressions in lung and immunohistochemistry of PCNA and SPC

**Figure 9:**
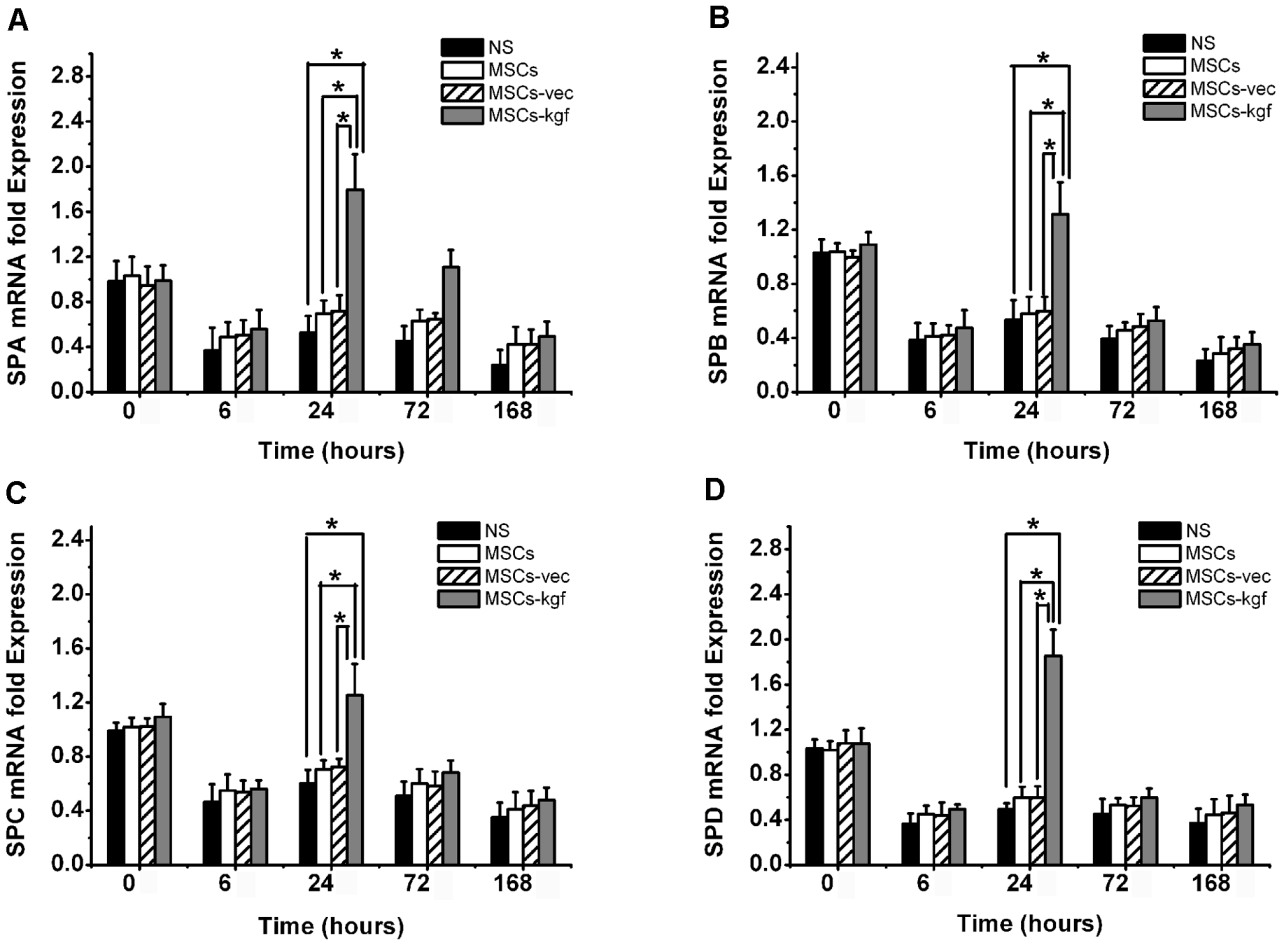
Analysis of SP mRNA expressions in ALI mice lungs. (A–D) mRNA levels of SPA, SPB, SPC and SPD after NS, MSCs, MSCs-vec or MSCs-KGF administration were quantitatively analyzed by real-time PCR, and the values were normalized to that of the NS group at 0 hours before LPS administration. Group comparisons were analyzed by a one-way ANOVA with Tukey-Kramer *post hoc* test. n = 5 per group. * *p*<0.05.

**Figure 10:**
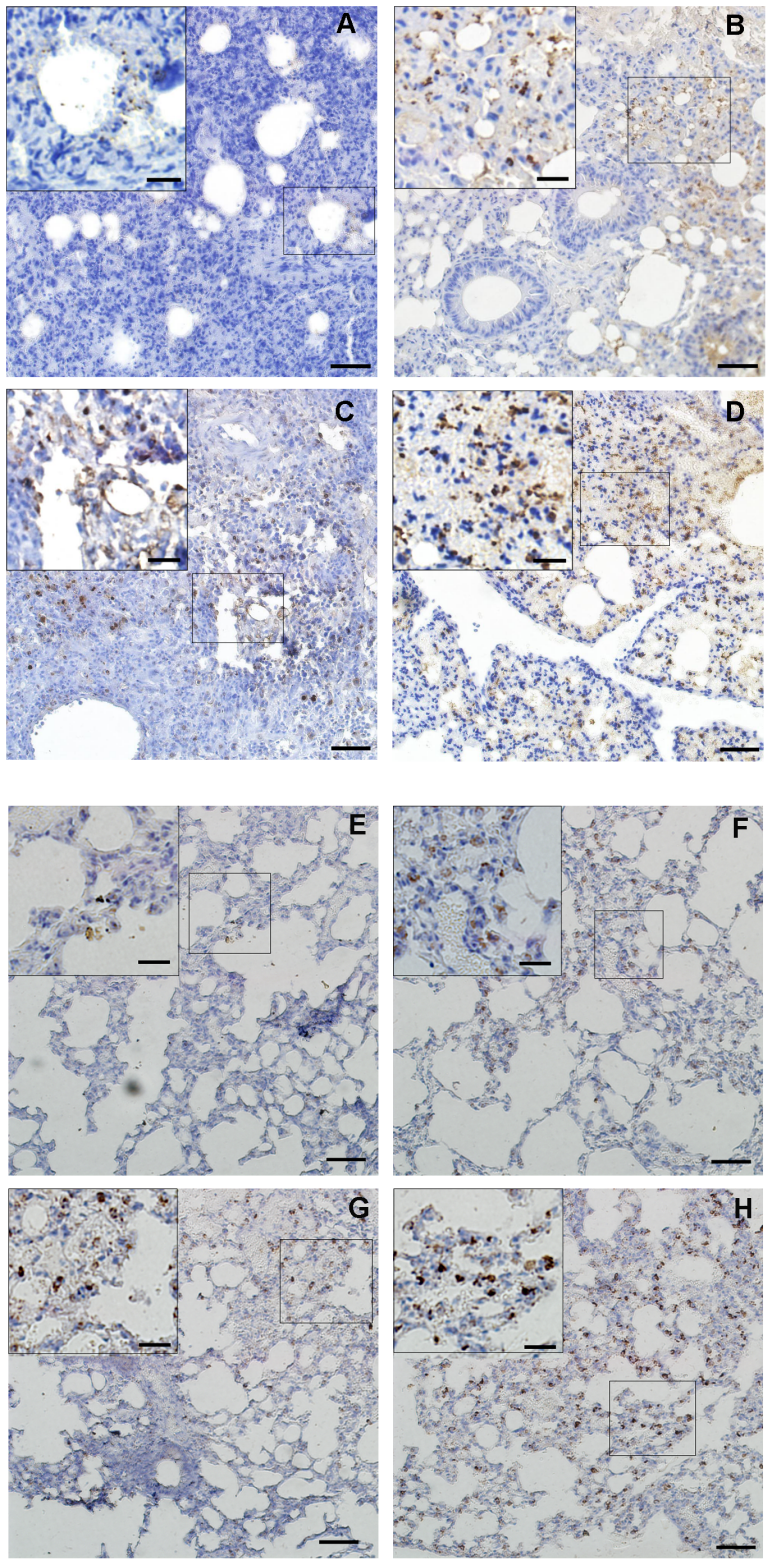
Immunohistochemistry of PCNA and SPC. Representative immunohistochemistry images for PCNA (A–D) and SPC (E–H) 72 hours after MSCs administration. More PCNA-positive cells and SPC-positive cells were present in the alveolar region in the MSCs-KGF group than that in the other three control groups, and most of these cells were not only positive for SPC but also positive for PCNA (A,E NS group; B,F MSCs group; C,G MSCs-vec group; D,H MSCs-KGF group; Scale bar =  50 µm; Inserts, scale bar =  20 µm).

We examined the SP mRNA expressions via real-time PCR with the same method used for the total KGF mRNA analysis. The mRNA expression of the four isotypes of SP showed the same trend. They were significantly increased in the MSCs-kgf group compared with the other three control groups at 24 hours after LPS administration (Figure 9. A, B, C, D, *p*<0.01 compared to NS group, *p*<0.05 compared to MSCs or MSCs-vec group). In addition, the immunohistochemistry showed that the percentages of both PCNA-positive and SPC-positive cells in the MSCs-kgf group were higher than that in the other three control groups (Figure 10). Furthermore, SP-C positive cells were observed in the alveolar regions, and most were also positive for PCNA.

## Discussion

In the present study, we found that transplanted KGF gene-modified MSCs were not only retained in the lung but also enhanced KGF expression in the lungs of LPS-challenged mice. We also demonstrated that MSC-mediated KGF gene delivery can significantly alleviate alveolar inflammation and permeability, while MSCs alone or MSCs-vec delivery has a lesser effect. Most importantly, our results revealed that MSCs-kgf transplantation can significantly improve the pathological changes that result from lung injury in mice.

ALI is initially characterized by the leakage of protein-rich edema fluid into the interstitium and alveolar space. Injury to the alveolar epithelium has been considered to be a key factor in the development of ALI [28]. Therefore, treatments that aim to repair or limit epithelial damage might become a key therapeutic strategy to accelerate recovery and decrease the mortality of ALI patients [5], [29]. KGF is a growth factor that has been shown to play an important role in pulmonary epithelial repair [30]. It can stimulate type II alveolar epithelial (AT2) cells to proliferate and increase alveolar epithelial fluid transport [5], [6], [31]. Accordingly, we attempted to over-express the KGF gene in the lung via MSC-based gene therapy to target the lung in this study. Lentiviral vectors were used to deliver transgenes because they could efficiently transfer genes to non-dividing cell, and the expression of the gene was likely to persist for the lifetime of the cell [29], [32]. Unlike the adenoviral vectors, lentiviral vectors do not causes inflammatory responses [32]. As shown in Figures 2.A, B and C, the increased levels of KGF protein and mRNA in MSCs proved the success of the gene transduction.

One of the foremost concerns of cell based-gene therapeutics is the retention of injected cells. MSCs have been shown to be more efficiently retained in injured tissues [12], [18]. To better detect MSCs *in vivo*, non-transduced MSCs were labeled with a cell tracker CM-Dil, and both MSCs-vec and MSCs-kgf were transduced with the tracer eGFP gene before they were intravenously injected into the mice. Both a confocal laser scanning microscope and immunostaining approaches were used to detect transplanted cells expressing GFP in the injured lung tissue at four time points. The results indicated that MSCs could mobilize to areas of inflammation and injury and were retained for a few days. Our observations are consistent with other reports [17], [18]. Moreover, we also detected KGF expression *in vivo*. We found that MSCs-kgf transplantation significantly increased the expression of the KGF gene in lung tissues, but not in the plasma (data not shown). This discrepancy may suggest that the beneficial effect of KGF in the lungs is local rather than systemic.

The time for MSCs-kgf transplantation is also very important to the treatment effect. Previous studies have reported various timings of MSCs administration, ranging from 15 min to 4 h. At present, an optimal timing of administration has not been determined for MSCs [11]. In this study, we administered MSCs-kgf two hours after LPS administration, which constituted an administration during the early stage of ALI. During this stage, the pro-inflammatory immune response has been initiated within the injured lung, and pro-inflammatory cytokines, such as TNF-α, have been released, which can stimulate the homing of MSCs to lung [33], [34]. In addition, KGF has been shown to dramatically reduce lung injury induced by acid administration [35], bleomycin administration [8] and a-naphthylthiourea [36]. However, the majority of these studies administered exogenous rhKGF a few days prior to the manifestation of illness. This experimental design deviates from clinical applications because such treatment would normally not be administered to the patient [37]. In the present work, we administered KGF via MSCs after injury onset and showed that KGF could enhance the therapeutic potential of MSCs. This effect may be due to the combination of MSCs and KGF, which not only allows the direct targeting of the lung for clinical intervention but also provides a site-specific source to release therapeutic KGF proteins and/or other cellular products by MSCs [15].

The main cause of ALI in this model was the inflammation induced by LPS. KGF gene-modified MSC transplantation significantly reduced the number of neutrophils in BALF and MPO activity, decreased the expression of the pro-inflammatory cytokines IL-1β and TNF-α in BALF and increased the expression of the anti-inflammatory cytokine IL-10. Furthermore, we also found that the function of the alveolar-capillary membrane was partly restored by the administration of MSCs-kgf, which was demonstrated by the reduced lung wet/dry ratio and BALF protein. Most importantly, MSC-based KGF gene therapy attenuated histopathological impairment and improved the lung injury scores. The observed effects might be attributable to the combined effects of MSC cell therapy and KGF gene therapy on ALI because MSCs alone or MSCs-vec transplantation showed few beneficial effects.

MSCs show great promise in the repair of injured tissues, and their efficacy in repairing lung injury has been assessed in a number of studies. MSC treatment has reportedly improved survival and lung inflammation upon intrapulmonary administration in an animal model of LPS-mediated ALI [12], [13]or upon systemic administration in experimental models of bleomycin-induced lung injury [38], [39]. We chose bone MSCs in the present study because they have several advantages. First, bones MSCs are easily accessible, and their isolation is straightforward. These cell populations can also be expanded to clinical scales in a relatively short period of time [40]. Second, bone MSCs are hypoimmunogenic because do not express major histocompatibility complex class II and costimulatory molecules on their surface [41]. This characteristic makes their clinical application more feasible because matching donors and recipients would be unnecessary. Moreover, bone MSCs can be receptive to transduction with integrating vectors and delivered to sites of lung injury to counteract the inflammatory process [42]. Recently, the transplantation of bone MSCs has been deemed safe and has been widely tested in clinical trials of diseases with encouraging results. Thus, this strategy may also be feasible for patients with ALI [43]–[45].

Finally, we attempted to determine possible mechanisms by which KGF ameliorated the LPS-induced ALI. We observed an increase in surfactant production in the MSCs-kgf group. The surfactant protein may improve gas exchange and lung compliance by preventing alveolar collapse in the early stages of ALI development [31], [46]. In addition, they also exhibit host-defense properties [31]. Moreover, an increase in the percentages of cells positive for PCNA and SP-C was also observed after MSCs-kgf transplantation. Because PCNA was a good index to estimate the proliferative activity [47] and SP-C is a marker of lung epithelial type II cells, this result supports the idea that the protection in ALI mice may be partly due to the mitogenic capacity of KGF on lung epithelial type II cells.

The current study features several limitations. First, we chose mice as the animal model to transplant syngeneic plus allogeneic gene-modified MSCs. The LPS-induced mouse model of ALI cannot fully reproduce the complexity of clinical ALI/ARDS in human patients. Furthermore, the prognosis of ALI mice did not significantly improve after MSCs-kgf transplantation, although Gupta et al. [12] obtained positive results after MSCs administration. This discrepancy may be related to the different study designs, including the dose of LPS (10 mg/kg vs. 5 mg/kg), the quantity of MSCs (5×10^5^/mice vs. 7.5×10^5^/mice), the route of MSCs administration (intravenous vs. intratracheal) and the timing of administration of MSCs (2 hours vs. 4 hours). Finally, the mechanisms that underlie this protective effect of KGF have not been completely elucidated. Future studies are needed to explore this issue.

In conclusion, we demonstrated MSC-based KGF gene therapy can attenuate pathological damage and alleviate the inflammatory response and pulmonary vascular permeability in an LPS-induced ALI mice model. These beneficial effects may be associated with KGF effects including type II alveolar epithelial cell proliferation and increased surfactant protein secretion. Thus, KGF-modified MSC therapy appears to be a promising strategy for ALI treatment.

## Supporting Information

Figure S1 **Characterization of MSCs isolated from C57BL/6 mice.** (A) MSCs were harvested from the marrow of femurs and tibiae of C57BL/6 mice. MSCs at passage 5 displayed spindle-like shape. (Scale bar =  200 µm; Inserts, scale bar =  50 µm). (B) Staining with oil red-O was used to detect MSCs that differentiated into adipocytes, identified by perinuclear red staining of fat globules. (Scale bar =  100 µm; Inserts, scale bar =  50 µm). (C) Staining with alizarin red was used to detect MSCs that differentiated into osteocytes, which form calcium nodes after 21 days in culture. (Scale bar =  200 µm; Inserts, scale bar =  50 µm). (D) The expression of CD90, CD29, CD34 CD31 and CD45 were evaluated by flow cytometry to MSCs and the results were CD31^−^CD34^−^CD45^−^CD29^+^ CD90^+^.
(TIF)

Figure S2 **Quantization of MSC persistence in the lung of LPS mice.** The number of GFP positive cells was determined from 20 randomly selected areas per high-power field (magnification, ×400) with Scion Image software in the lung at fourt time points after MSCs-vec or MSCs-kgf transplantation. Group comparisons were analyzed by Student's t-test. n = 5 per group.
(TIF)
