# Supplementary figures and images for: Keratinocyte Growth Factor Gene Delivery via Mesenchymal Stem Cells Protects against Lipopolysaccharide-Induced Acute Lung Injury in Mice
Source: PLoS One. 2013 Dec 18;8(12):e83303. doi: 10.1371/journal.pone.0083303 (PMC3867420; doi:10.1371/journal.pone.0083303)

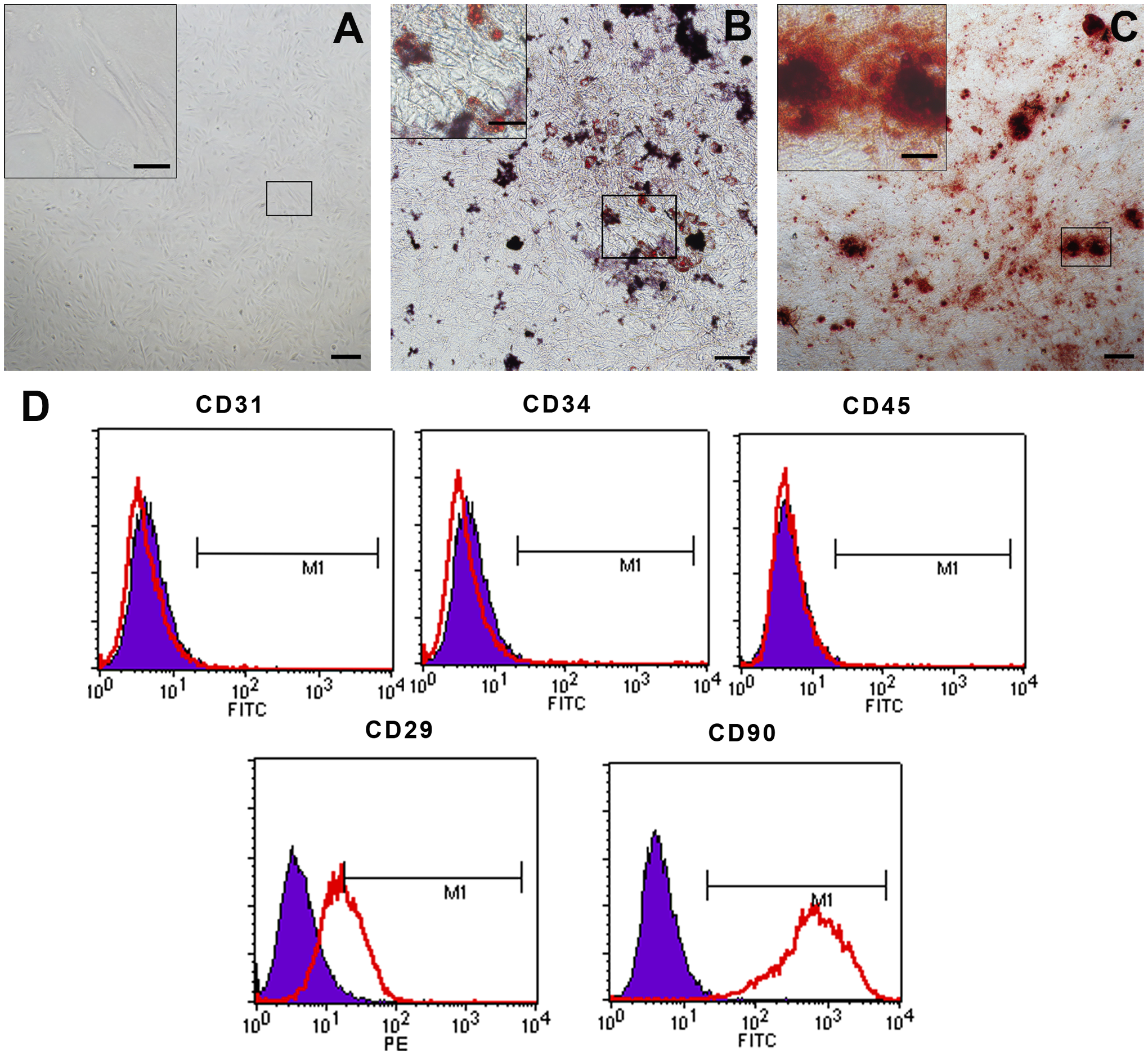

Supplement: Figure S1 — Characterization of MSCs isolated from C57BL/6 mice. (A) MSCs were harvested from the marrow of femurs and tibiae of C57BL/6 mice. MSCs at passage 5 displayed spindle-like shape. (Scale bar = 200 µm; Inserts, scale bar = 50 µm). (B) Staining with oil red-O was used to detect MSCs that differentiated into adipocytes, identified by perinuclear red staining of fat globules. (Scale bar = 100 µm; Inserts, scale bar = 50 µm). (C) Staining with alizarin red was used to detect MSCs that differentiated into osteocytes, which form calcium nodes after 21 days in culture. (Scale bar = 200 µm; Inserts, scale bar = 50 µm). (D) The expression of CD90, CD29, CD34 CD31 and CD45 were evaluated by flow cytometry to MSCs and the results were CD31−CD34−CD45−CD29+ CD90+. (TIF) [file pone.0083303.s001.tif]
